# Development of a new paradigm model for deciphering action mechanism of Danhong injection using a combination of isothermal shift assay and database interrogation

**DOI:** 10.1186/s13020-024-01017-6

**Published:** 2024-10-05

**Authors:** Tianxiang Wang, Changmei Yang, Yuxiang Tang, Ke Wen, Yuxin Ma, Yuling Chen, Zhiqiang Li, Yujiao Zhao, Songbiao Zhu, Xianbin Meng, Sijing Du, Zelong Miao, Wei Wei, Haiteng Deng

**Affiliations:** 1 MOE Key Laboratory of Bioinformatics, Center for Synthetic and Systematic Biology, School of Life Sciences, Tsinghua University, Beijing, 100084 People’s Republic of China; 2 Chinese Institutes for Medical Research, Beijing, China; 3 Wangjing hospital of China Academy of Chinese Medical Sciences, Beijing, China

**Keywords:** Isothermal shift assay (iTSA), TCM, DHI, Target identification, ADK, ALDH1B1

## Abstract

**Background:**

Identification of active components of traditional Chinese Medicine (TCM) and their respective targets is important for understanding the mechanisms underlying TCM efficacy. However, there are still no effective technical methods to achieve this.

**Methods:**

Herein, we have established a method for rapidly identifying targets of a specific TCM and interrogating the targets with their corresponding active components based on Isothermal Shift Assay (iTSA) and database interrogation.

**Results:**

We optimized iTSA workflow and identified 110 targets for Danhong injection (DHI) which is used as an effective remedy for cardiovascular and cerebrovascular diseases. Moreover, we identified the targets of the nine major ingredients found in DHI. Database interrogation found that the potential targets for DHI, in which we verified that ADK as the target for salvianolic acid A and ALDH1B1 as the target for protocatechualdehyde in DHI, respectively.

**Conclusion:**

Overall, we established a novel paradigm model for the identification of targets and their respective ingredients in DHI, which facilitates the discovery of drug candidates and targets for improving disease management and contributes to revealing the underlying mechanisms of TCM and fostering TCM development and modernization.

**Graphical Abstract:**

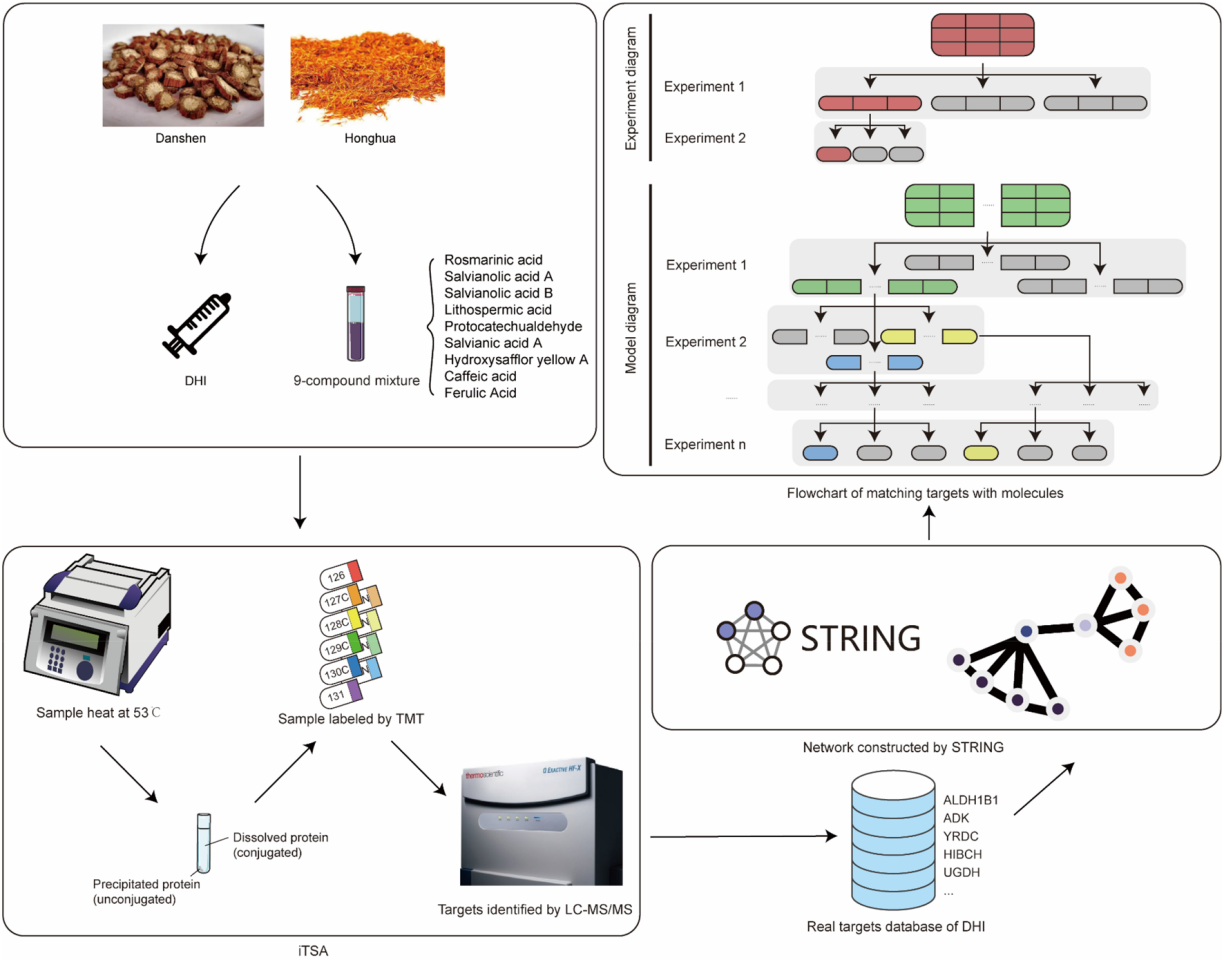

**Supplementary Information:**

The online version contains supplementary material available at 10.1186/s13020-024-01017-6.

## Highlights

1. Utilizing the innovative iTSA approach, we identified the targets of DHI and their respective active components.
2. Results demonstrated that salvianolic acid A in DHI targeted ADK while protocatechualdehyde targeted ALDH1B1 to exercise the functions of DHI.
3. The established workflow can facilitate a deeper understanding of action mechanisms of TCM.

**Supplementary Information:**

The online version contains supplementary material available at 10.1186/s13020-024-01017-6.

## Introduction

Traditional Chinese medicine (TCM) is extensively utilized in treating various clinical conditions including cardiovascular and cerebrovascular diseases [1], Alzheimer’s disease [2], and chronic gastritis [3], allergic diseases [4] and so on. The complexity of TCM stems from its multi-ingredients and multi-targets, which makes it challenging to elaborate its pharmacological mechanisms. The molecular ingredients of TCM represent a vast natural library of potential drug molecules. TCM formulations typically comprise multiple active ingredients that target multiple proteins and biological pathways, thus serving as a rich source for identifying lead compounds for drug discovery. The exploration and identification of effective active ingredients from TCM holds significant importance. Therefore, developing a robust method for identifying targets of TCM is invaluable for deciphering the mechanistic underpinnings of its therapeutic effects and discovering novel drugs.

Two major approaches have been utilized in drug discovery: one is the target-based drug discovery (TDD), and the other is phenotypic drug discovery (PDD) [5, 6]. Each approach possesses distinct advantages and drawbacks. TDD offers the advantage of known targets and mechanisms, such as specific mutations in cancers [6]. However, many diseases involve multi-targets and multi-mechanisms such as Alzheimer’s disease [7], obesity, cardiovascular and cerebrovascular diseases. The drugs discovered through TDD approach often find it difficult to treat these diseases. In this context, PDD involves screening drugs based on phenotypic changes, without prior knowledge of specific targets or mechanisms involved [8, 9]. With the ongoing advancements in target identification technologies, PDD often holds an edge in discovering new drugs [8, 10]. Natural products derived from TCM serve as a promising library for PDD strategy [11–13]. Due to TCM’s efficacy across numerous diseases, coupled with its notable advantage in the field of PDD, the significance of PDD in advancing TCM research and discovering novel therapeutics for diverse diseases is profound.

Danhong injection (DHI), composed of Danshen (*Salvia miltiorrhiza*) and Honghua (*Carthamus tinctorius*), is clinically administered for the treatment of cardiovascular and cerebrovascular diseases, including coronary heart disease, angina pectoris, and ischemic stroke [14]. Both Danshen and Honghua exhibit various pharmacological activities, such as cardioprotective effects, anti-atherosclerotic and anti-inflammatory properties [1, 15]. Main ingredients of DHI include Rosmarinic acid, Salvianolic acid A, Salvianolic acid B, Lithospermic acid, Protocatechualdehyde, Salvianic acid A, Hydroxysafflor yellow A, Caffeic acid, Ferulic Acid [14, 16]. Several of these ingredients have been reported efficacy in treating cardiovascular and cerebrovascular diseases [17, 18].

Network pharmacology analysis (NPA) is commonly used for investigating the targets and mechanisms of TCM [19]. It mainly relies on database resources and computational methods to predict key ingredients and targets for diseases. However, these predictions carry inherent limitations and uncertainties. While network pharmacology can provide valuable clues and hypotheses, further experimental validation is required to ascertain the actual targets of molecules. It is difficult to accelerate the modernization of TCM solely through network pharmacology. Therefore, for a comprehensive exploration of actual targets of TCM mixture and the mechanisms of disease treatment, a better research paradigm is crucial.

Isothermal Shift Assay (iTSA) is a highly efficient method used for the identification of small molecule targets [20]. This method is based on the enhanced thermal stability resulting from the binding of small molecules to their targets, which consequently leads to a higher abundance. In contrast to thermal proteome profiling (TPP) [21], iTSA exhibits a higher target count [20]. Moreover, the iTSA method offers more replicates, enhanced reproducibility, and simplified operation compared to TPP. Therefore, for the modernization of TCM, iTSA-based method can identify targets for TCM mixture or active ingredients, thereby significantly contributing to the advancement of PDD.

Herein, we conducted iTSA to identify the targets of DHI mixture. It is noteworthy that numerous targets of DHI have been reported to be associated with cardiovascular and cerebrovascular diseases. Importantly, unreported targets may serve as potential therapeutic targets. Furthermore, we also analyzed DHI targets alongside with the ischemic stroke targets, revealing potential impacts on the HIF-1 signaling pathway and metabolic pathways. Moreover, we also conducted iTSA to identify the targets of the 9 main ingredients of DHI, uncovering common targets namely ALDH1B1, HIBCH, YRDC, BLVRA, GMDS, ADK, AKR7A2, and UGDH. Furthermore, we have identified ALDH1B1 as a target of protocatechualdehyde and ADK as a target of salvianolic acid A. Overall, we have established a model for studying targets and mechanisms of TCM mixture or active ingredients. This research model facilitates the discovery of new drugs and targets for diseases treatment, as well as provides a deeper understanding of the mechanisms how TCM exerts its therapeutic effects.

## Materials and methods

### Cell preparation and culture, reagents

AC16 cells and human umbilical vein endothelial cells (HUVECs) (purchased from the cell bank of the Chinese Academy of Sciences), were cultured in DMEM (Wisent, Montreal, Canada). The culture medium was supplemented with 10% fetal bovine serum (FBS) (PAN-Biotech, Germany) and 1% penicillin/streptomycin (Wisent, Montreal, Canada). Cells were cultured in a humidified incubator containing 5% CO_2_ at 37 °C.

Danhong injection (batch number 20081023, Shandong Province, China) was purchased from Shandong Buchang Pharmaceutical. Rosmarinic acid (Cat# IR0140), salvianolic acid A (Cat# IS0540), salvianolic acid B (Cat# IS1940), ferulic acid (Cat# IF0270), caffeic acid (Cat# IC1040), lithospermic acid (Cat# IL0420) and hydroxysafflor yellow A (Cat# IH0140) were purchased from Solarbio (China), protocatechualdehyde (Cat# T3018) and salvianic acid A (Cat# T3227) were purchased from TargetMol (USA).

### Isothermal shift assay (iTSA)

After cell collection, the cells were washed twice with PBS, then resuspended in lysis buffer (PBS containing protease inhibitors cocktail and 1.5 mM MgCl_2_). The cells were disrupted using a glass homogenizer on ice. Subsequently, the cell lysate was evenly divided into two portions, with one portion receiving a solvent and the other receiving the DHI (volume ratio is 100:1) or molecules (100 μM). After incubation at room temperature for 15 min, the lysate was further divided into 5 equal aliquots and heated at 53 °C for 3 min. Then, the lysate was incubated at 25 °C for 3 min. Following the heat treatment, the samples were incubated with a final concentration of 0.4% NP40 and 250 U/mL Benzonase (Sigma, Cat# E1014) for 1 h at 4 °C on a shaking platform, aiming to effectively extract membrane-bound and DNA-bound proteins. Next, the sample will be subjected to ultracentrifugation at 100,000 g, 4 °C for 20 min, and then the supernatant will be taken for quantitative proteomic analysis with tandem mass tag (TMT) labeling or Western blot analysis.

### Sample preparation for tandem mass tag (TMT) quantitative proteomic analysis

The sample preparation will be conducted as previously described [22]. Briefly, for the preprocessing of TMT-labeled proteomic samples from the supernatant obtained through the iTSA method, acetone is first added in a volume ratio of 1:5 and precipitated overnight at − 30 °C. Subsequently, the acetone is removed by centrifugation at 10,000 g, 4 °C for 10 min. The precipitate is then redissolved in 8 M urea. After measuring the concentration, approximately 100 ug of protein solution from each sample is taken and subjected to reduction and alkylation treatment using DTT and chloroacetamide. Digestion is then performed with trypsin at a ratio of 40:1 for 16 h at 37 °C. The resulting peptides are desalted and labeled with TMT reagents (Thermo Scientific, Cat# 90110). The control group with five replicates is labeled with 126, 127N, 127C, 128N, and 128C, while the treatment group with five replicates is labeled with 129N, 129C, 130N, 130C, and 131. Subsequently, LC–MS/MS analysis is carried out.

LC–MS/MS analysis was performed on a Q Exactive HF-X mass spectrometer (Thermo-Fisher Scientific) online coupled to a nano-HPLC system (Thermo-Fisher Scientific) with a procolumn connected to a C18 column (Waters). The raw mass spectrometry data were searched against the Homo Sapiens database by the Proteome Discoverer 2.3 software. Raw data of LC–MS/MS have been uploaded to the iProx website (https://www.iprox.org). The accessible number is IPX0008403000.

### Proteomic data analysis

Proteome data were filtered by the criteria shown below. (1) Sum PEP Score >  = 5. (2) Unique Peptides >  = 1. (3) Proteins contain any missing value were deleted. (4) Proteome data were normalized by total abundance, Internal Reference Scaling (IRS) and Trimmed mean of M-values normalization (TMM), sequentially.

Filtered data were subsequently subject to fold change (FC) calculation and Student’s t-test. FC threshold for distinguishing target proteins and others was determined by variance-coverage plot. PCA was used to visualize separation of drug treated and untreated proteome. p-value < 0.05 and FC > 1.3 proteins were considered as targets. Then Fisher’s exact test was performed to find enriched gene sets (Gene Ontology [23]) or pathways (KEGG [24]).

### Bioinformatic analysis

Bioinformatic analysis processes can be mainly divided into the following parts.

- (1) Gene set acquisition.

A total of 3559 Ischemic Stroke (IS)-related proteins were obtained from Genecards [25] (https://www.genecards.org/), Drugbank [26] (https://go.drugbank.com/), PharmGKB [27] (https://www.pharmgkb.org/), OMIM (Online Mendelian Inheritance in Man, OMIM^®^. McKusick-Nathans Institute of Genetic Medicine, Johns Hopkins University (Baltimore, MD). World Wide Web URL: https://omim.org/). Venn diagram (https://bioinformatics.psb.ugent.be/webtools/Venn/) was plotted to show the intersection of IS-related proteins and DHI targets.

- (2) Enrichment analysis.

GO background gene set was obtained from UniProt [28] (https://www.uniprot.org/). KEGG background gene set was obtained by KEGG API [24]. Enrichment analysis was then performed using the R package clusterProfiler [29].

- (3) Protein–protein interaction (PPI) network construction.

Target protein interaction network was constructed by STRING [30] (https://string-db.org/) and subsequently formatted by Cytoscape v3.10.1 [31].

- (4) Protein-related disease retrieval.

The intersection of targets between 9 mixture and IS yielded 8 proteins. The association of these 8 proteins with diseases was obtained from the DisGeNET (v7.0) database [32].

### Cellular thermal shift assay (CETSA)

After cell collection, the cells were washed twice with PBS, then resuspended in lysis buffer (PBS containing protease inhibitors cocktail and 1.5 mM MgCl_2_). The cells were disrupted using a glass homogenizer on ice. Subsequently, the cell lysate was evenly divided into two portions, with one portion receiving a solvent and the other receiving the drug. After incubation at room temperature for 15 min, the lysate was further divided into 10 equal aliquots and heated at different temperatures for 3 min each (37 °C, 40.6 °C, 43.7 °C, 47.5 °C, 51.2 °C, 54.5 °C, 57.9 °C, 61.5 °C, 64 °C, 67 °C). Finally, the lysate was incubated at 25 °C for 3 min.

### Western blotting analysis

Equal volumes of proteins were separated by 12% sodium dodecyl sulfate–polyacrylamide gel electrophoresis (SDS-PAGE) and electro-transferred onto a polyvinylidence difluoride (PVDF) membrane. Primary rabbit anti-ALDH1B1 (Cloud-Clone, Wuhan, China), rabbit anti-ADK (Abcam, Cambridge, UK), rabbit anti-actin (ABclonal, Wuhan, China), and secondary anti-rabbit HRP-IgG antibodies (Cell Signaling Technology, Danvers, USA) were used for immunoblotting.

## Results

### Targets identification of DHI by iTSA

**Fig. 1:**
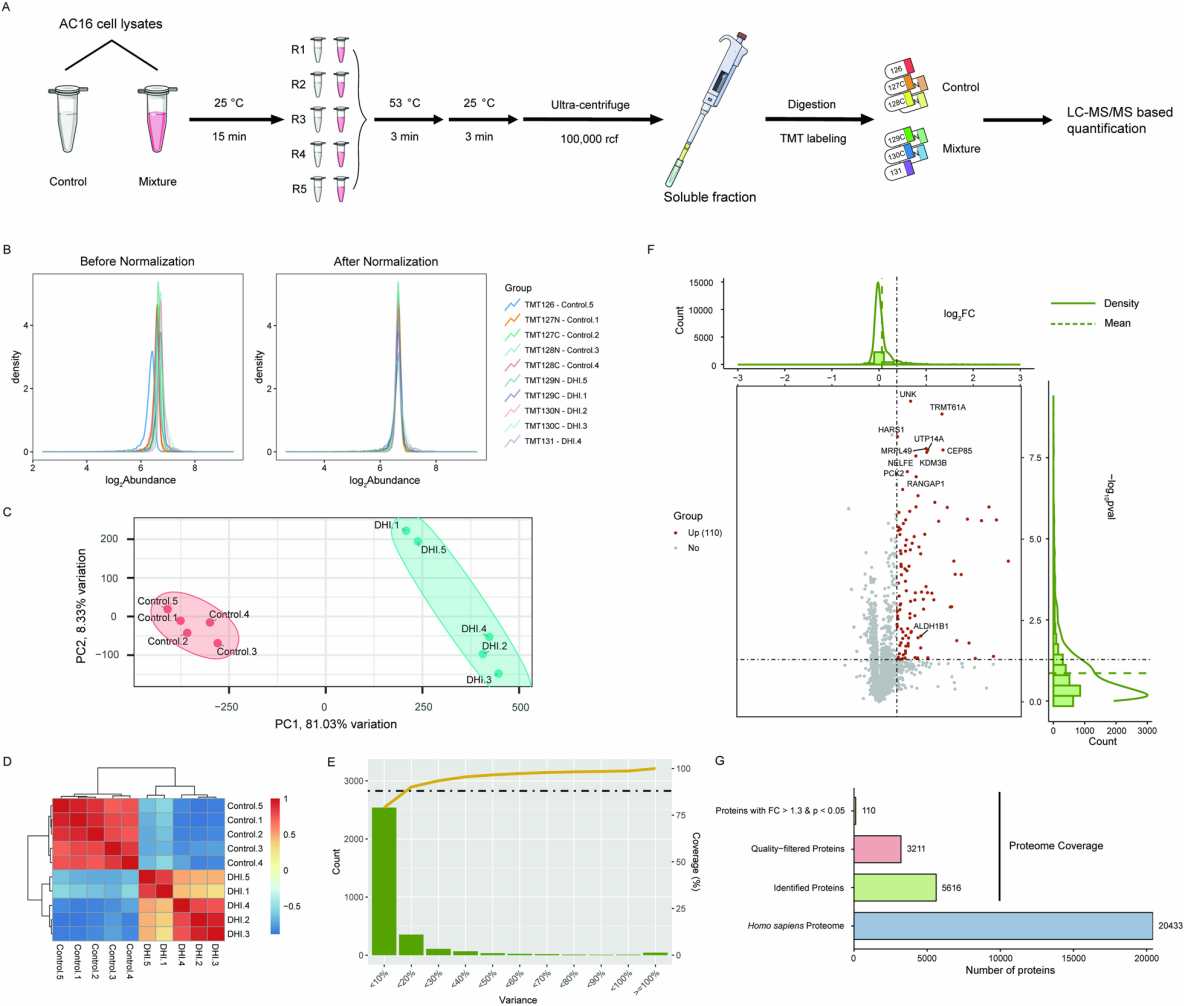
Targets identification of Danhong injection (DHI) by Isothermal Shift Assay (iTSA). **A** Flow chart of Isothermal Shift Assay (iTSA). In brief, the protocol comprises subjecting samples, either untreated or drug-treated, to a thermal challenge at 53 °C to precipitate proteins. Subsequently, ultra-centrifugation is utilized to separate the precipitate from the liquid phase. Following this separation, proteomic analyses are performed using Tandem Mass Tag (TMT) labeling to identify target proteins, which typically exhibit increased abundance in drug-treated samples. **B** The proteomics results were normalized using the Trimmed Mean of M-values (TMM) method. **C**, **D** The results of Principal Component Analysis (PCA) and correlation plot of validated proteins. **E** The threshold (Fold Change > 1.3) of target proteins was determined by Variance-Coverage plot. **F** Volcano plot showed the targets. **G** Overview of proteome data. We identified 5616 proteins and obtained 110 targets from 3211 quality-filtered proteins

To identify the targets of DHI, we optimized iTSA workflow using AC16 cells. Isothermal Shift Assay (iTSA) was performed by incubating cell lysates with vehicle or DHI for 15 min at 25 °C. The assay consisted of 5 control groups and 5 experimental groups, which were heated at a temperature of 53 °C for a duration of 3 min (Fig. 1A). Subsequently, quantitative proteomics with tandem mass tag (TMT) 10-plex reagents labeling was conducted followed by LC–MS/MS (Fig. 1A). Proteomic results were normalized using the Trimmed Mean of M-values (TMM) method (Fig. 1B). Principal Component Analysis (PCA) and correlation plot of identified proteins indicated the proteomic results exhibit good reproducibility (Fig. 1C, D). To identify the binding proteins of DHI, the Variance-Coverage plot was generated (Fig. 1E), with the threshold cutoff set at 88% coverage [33]. Based on this result, proteins that had a fold change > 1.3 with Student’s *t*-test p < 0.05 were considered as targets proteins upon the addition of DHI (Fig. 1F). Among the 5616 proteins identified, 110 proteins were listed as the DHI-targeted proteins (Fig. 1G).

**Fig. 2:**
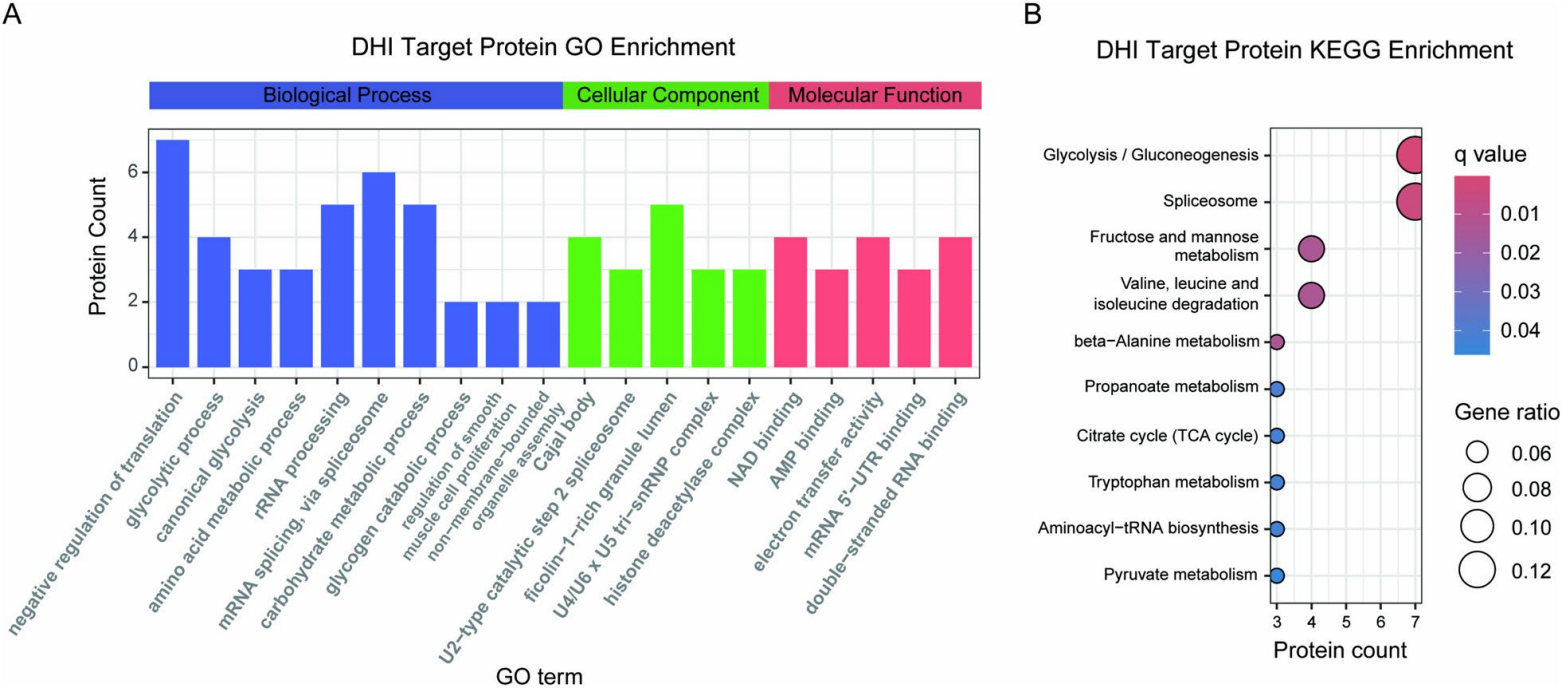
Functional analysis of DHI target proteins. **A** A bar plot depicting GO enrichment analysis, with Biological Process (BP), Cellular Component (CC), and Molecular Function (MF) enrichment analysis indicated by blue, green, and red bars, respectively. **B** Top 10 results from the KEGG enrichment analysis of 110 targets

Subsequently, Gene Ontology (GO) analysis was conducted on the 110 targets of DHI to delineate the biological processes (BP) and molecular functions (MF) they are involved in. BP terms of the 110 targets predominantly encompassed negative regulation of translation, rRNA processing, glycolytic process and electron transport chain. MF terms are mainly involved in NAD binding, electron transfer activity (Fig. 2A). Moreover, Kyoto encyclopedia of genes and genomes (KEGG) pathway analysis revealed enrichment in glycolysis/gluconeogenesis, spliceosome, fructose and mannose metabolism pathways (Fig. 2B). Collectively, our iTSA data indicated that DHI had multiple targets and may affect various metabolic pathways (Fig. 2B).

### Correlations between DHI targets and proteins involved in ischemic stroke

**Fig. 3:**
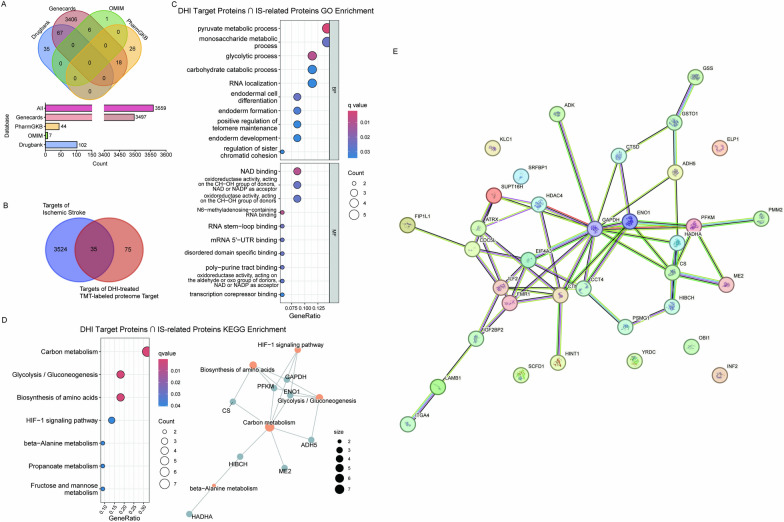
Analysis of the correlation between DHI targets and cardiovascular and cerebrovascular diseases. **A** Venn diagram of IS-related targets in 4 databases (DrugBank, GeneCards, OMIM, and PharmGKB). A total of 3559 targets were obtained. **B** The Venn diagram compares DHI targets with those related to IS, revealing 35 shared targets. **C** GO enrichment analysis of the 35 common targets between DHI and IS. **D** KEGG enrichment analysis of the 35 common targets between DHI and IS. **E** Protein–protein interaction (PPI) network of 35 targets constructed by STRING

DHI is primarily used for the treatment of cardiovascular and cerebrovascular diseases. Taking ischemic stroke (IS) as an example, we investigated the mechanism of DHI in treating IS. IS is a leading cause of global mortality [34]. Although DHI has been used clinically to treat IS, its mechanisms of action remain elusive. Therefore, we identified a total of 3559 genes associated with IS from the DrugBank, GeneCards, OMIM, and PharmGKB databases (Fig. 3A). Then a venn diagram analysis was performed to compare the targets of DHI identified by iTSA with those related to IS (Fig. 3B). The results showed that among the 110 targets of DHI, 35 targets were reported and statistically validated in the databases, while 75 potential targets did not match any known targets.

To reveal the mechanisms of DHI in IS treatment, we analyzed the BP and MF terms of the 35 overlapped targets with GO analysis. The results indicated that BP were mainly involved in pyruvate metabolic process, monosaccharide metabolic process, glycolytic process, carbohydrate catabolic, while molecular functions were mainly involved in NAD binding, oxidoreductase activity (Fig. 3C). Furthermore, KEGG pathway analysis revealed enrichment in carbon metabolism, glycolysis/gluconeogenesis, biosynthesis of amino acids, and the HIF-1 signaling pathways (Fig. 3D). Notably, HIF-1 has been reported as a crucial regulator in potential therapeutic approaches to IS [35]. Our results further highlighted GAPDH, PFKM, and ENO1 as three common targets involved in the HIF-1 signaling pathway. Moreover, Protein–Protein Interaction (PPI) Networks constructed by STRING demonstrated the interactions among the 35 common targets between DHI and IS (Fig. 3E). These results indicate that the targets of DHI, identified via the utilization of iTSA, provide significant insights into the mechanisms of DHI’s treatment of diseases.

### Target identification for 9 major ingredients of DHI using iTSA

**Fig. 4:**
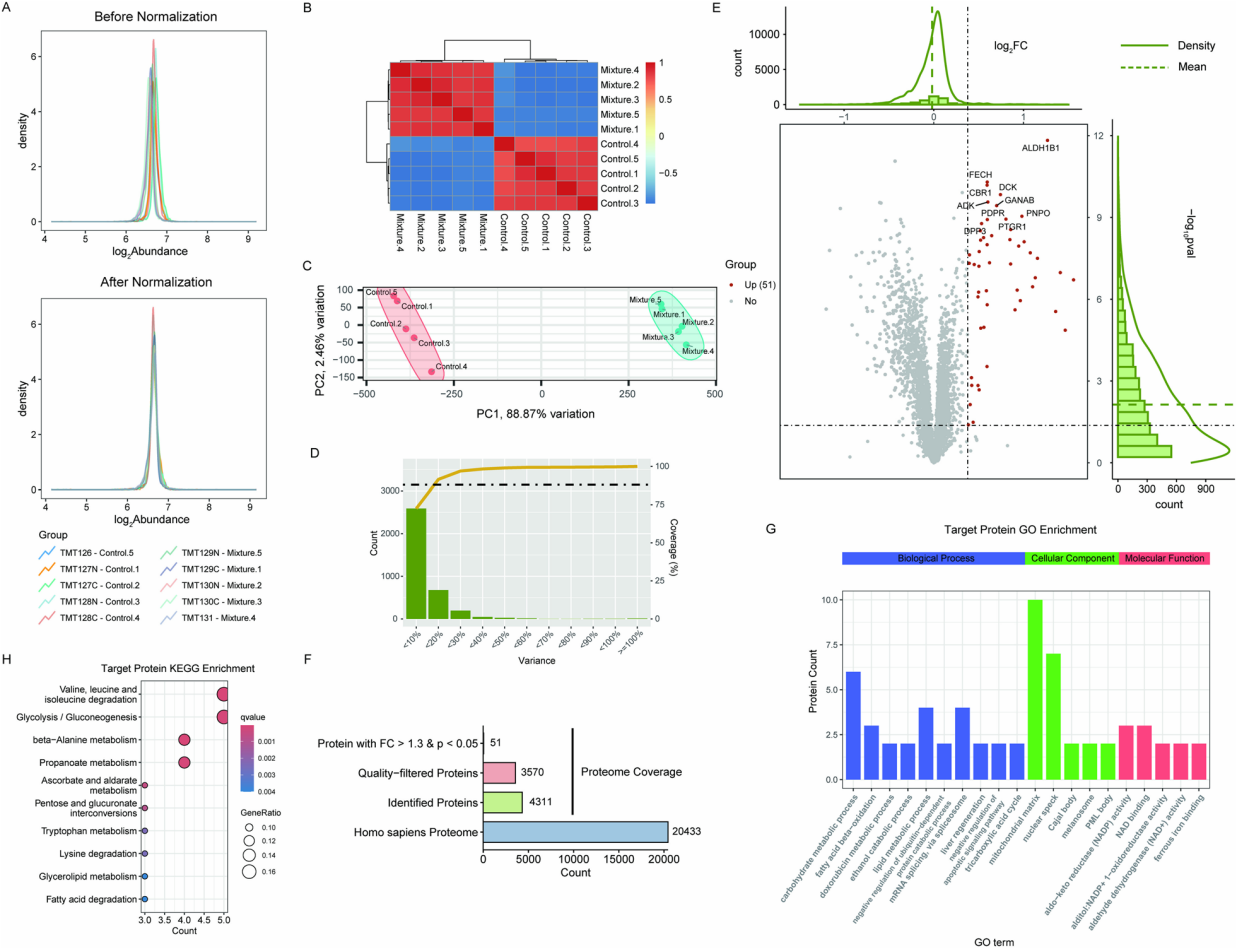
Target identification of 9 compounds mixture by iTSA. **A** Normalization of proteome data was conducted using Trimmed Mean of M-values (TMM) methods. The legend indicates the correspondence between TMT labels and experimental groups. **B**, **C** Correlation plot and PCA analysis were performed on the validated proteins to assess their relationships and variabilities. **D** The threshold was determined using a Variance-Coverage plot, with a Fold Change criterion of greater than 1.3. **E** A volcano plot was utilized to visually represent and identify the significant targets. **F** An overview of the proteome data revealed that 4311 proteins were identified. After quality filtering, 3570 proteins remained, from which 51 targets were ultimately obtained. **G** A bar plot depicting GO enrichment analysis, with Biological Process (BP), Cellular Component (CC), and Molecular Function (MF) enrichment analysis indicated by blue, green, and red bars, respectively. **H** KEGG enrichment analysis of the targets was conducted, and the top 10 results are presented

The main ingredients are commonly regarded as the active molecules in TCM. Hence, we also conducted iTSA to identify the targets of main ingredients of DHI. From previously published studies, Rosmarinic acid, Salvianolic acid A, Salvianolic acid B, Lithospermic acid, Protocatechualdehyde, Salvianic acid A, Hydroxysafflor yellow A, Caffeic acid, Ferulic Acid are main ingredients of DHI [14, 16] (Supplementary Table 1). These 9 compounds were mixed followed by iTSA analysis. We processed the data using the same method as before. Briefly, we normalized the proteomics results using the TMM methods (Fig. 4A, B). Then, correlation analysis and PCA analysis were conducted to affirm data quality (Fig. 4C). Variance-Coverage plot was generated to determine the threshold (fold change > 1.3) of target proteins (Fig. 4D). From the analysis, a total of 4311 proteins were identified in which 51 target proteins were obtained from 3570 quality-filtered proteins (Fig. 4E, F). Subsequently, we performed GO to identify the BP and MF associated with the main ingredients. BP terms were mainly involved in the carbohydrate metabolic process, lipid metabolic process and mRNA spliceosome. MF terms were mainly involved in aldo–keto reductase (NADP) activity and NAD binding (Fig. 4G). KEGG pathway analysis unveiled enrichment in valine, leucine and isoleucine degradation, glycolysis/gluconeogenesis, beta-alanine metabolism and propanoate metabolism pathways (Fig. 4H).

### Establishment of targets-ingredient correlations

**Fig. 5:**
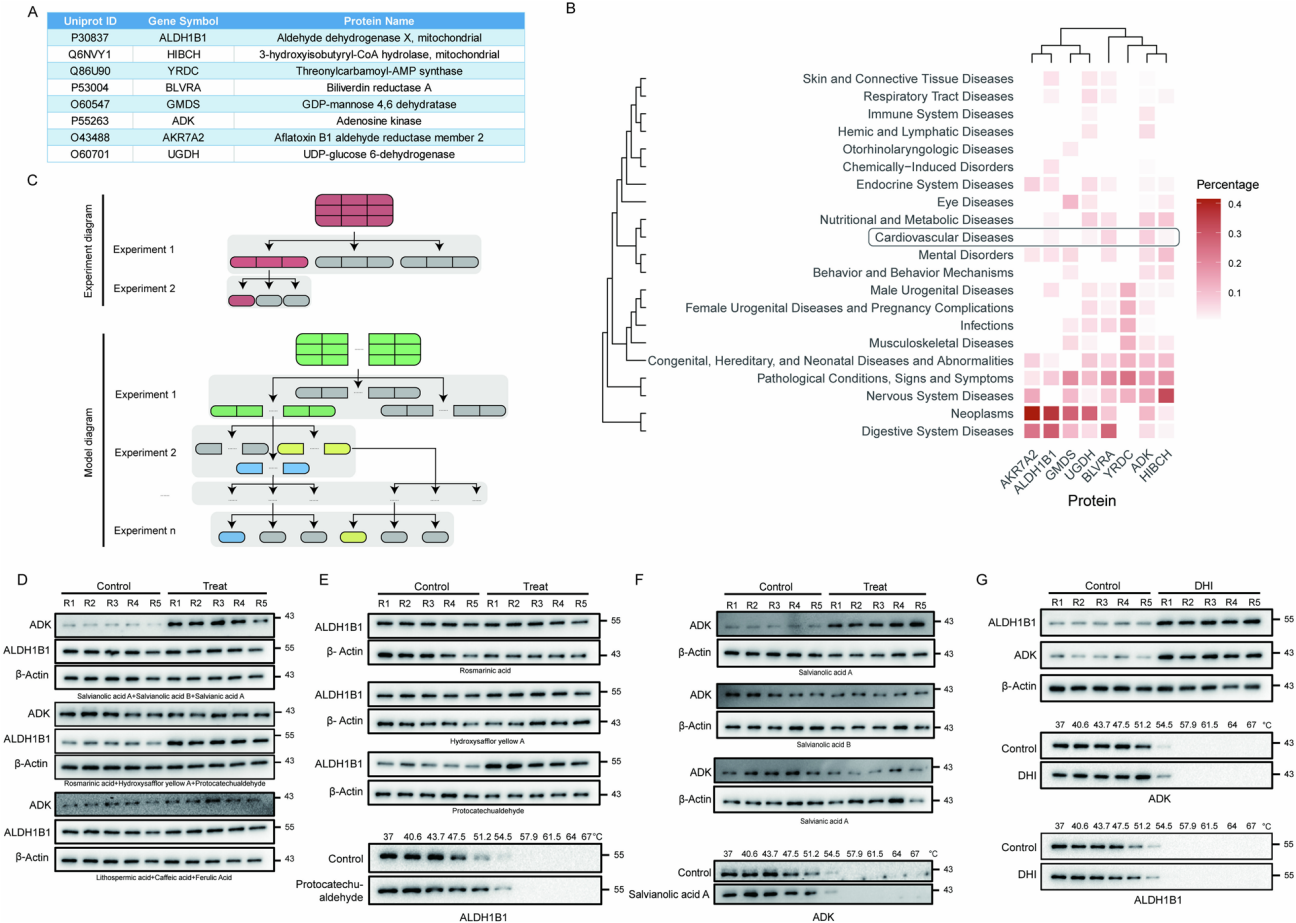
A ligand fishing workflow for interrogating the targets with corresponding binding molecules. **A** Common targets between DHI and 9 ingredients. **B** A heatmap of the correlation between these common targets and diseases. **C** A flowchart for fishing out the corresponding small molecules. **D**–**G** Western blot analysis was performed to fish out small molecules

Based on the targets identified for the nine ingredients, we obtained 8 common targets with DHI, namely ALDH1B1, HIBCH, YRDC, BLVRA, GMDS, ADK, AKR7A2 and UGDH (Fig. 5A). To investigate the correlation between targets and diseases, we conducted bioinformatics analysis, indicating that these targets are involved in various diseases, such as digestive system diseases, neoplasms and cardiovascular diseases (Fig. 5B). Our primary focus is on their roles in cardiovascular diseases, the result uncovered involvement of ADK, BLVRA, ALDH1B1, UGDH, and HIBCH. For instance, cardiomyocyte ADK deletion was reported ameliorating myocardial ischemia/reperfusion injury and ADK inhibition achieve sustained cardioprotection [36–38]. ALDH1B1, a mitochondrial ALDH isoform, possesses the capability to oxidize various substrates. Using ADK and ALDH1B1 as examples, we aimed to identify the corresponding small molecules that bind to these targets.

We have devised an assay to identify the ligands/molecules that bind to a specific target, as outlined in Fig. 5C. This workflow consists of three key steps: (1) Small Molecule Grouping: we divided nine small molecules into three groups to simplify the screening process and efficiently identify target-binding molecules. (2) Initial Screening, we conducted initial screening to determine the group containing the binding molecule. (3) Validation, we performed precise screening by exposing individual molecules from the identified group to the target protein, ultimately identifying the specific small molecule that binds to the target protein. Herein, the nine molecules were divided into three groups: (a) Salvianolic acid A + Salvianolic acid B + Salvianic acid A, (b) Rosmarinic acid + Hydroxysafflor yellow A + Protocatechualdehyde, and (c) Lithospermic acid + Caffeic acid + Ferulic acid. Through the first experiment, we identified (a) and (b) groups of molecules binding to ADK and ALDH1B1, respectively (Fig. 5D). Subsequent experiments were conducted to identify the specific molecules that bind to each target, with cellular thermal shift assay (CETSA) utilized for confirmation (Fig. 5E, F). Similarly, single-temperature experiments and CETSA were performed on DHI for confirmation (Fig. 5G). Therefore, we applied this workflow and identified salvianolic acid A as the molecule that binds to ADK, and protocatechualdehyde as the molecule that binds to ALDH1B1. These results strongly affirm the feasibility and efficacy of our method. By extending this assay, a larger quantity of known small molecules (eg. n = 200 or even more) could be mixed to identify their targets and fish out their respective binding small molecules quickly. Through this approach, we can precisely identify which small molecule binds to the target protein.

## Discussion

TCM such as Yunnan Baiyao and Pien Tze Huang has demonstrated remarkable efficacy [39, 40]. However, owing to its complex composition, TCM presents a challenge with its multiple targets and diverse mechanisms of action. Therefore, improved techniques are imperative for target identification and mechanism analysis in disease treatment. Proteomics emerges as a promising avenue in the target identification of small molecule [41]. Techniques such as TPP and iTSA have been developed based on the principle that protein thermal stability will be enhanced after binding to small molecule [20, 42], while LiP-MS technology relies on structural changes resulting from the interactions between small molecules and proteins [43]. These proteomic techniques offer profound insights into the targets and mechanisms of TCM, thereby fostering its advancement and clinical implementation. Previous studies have reported that the iTSA method identifies a larger number of targets compared to TPP attributed to the higher number of replicates in iTSA and consequently lower false-positive rates [20]. In this study, we also observed that iTSA can aptly reflect minor changes (Fig. 5D–G), whereas it is a feat challenging to achieve the same with TPP, due to operational complexities and disparities in data processing. The augmented replicates in iTSA yield a more robust and reliable dataset, enabling more accurate identification of targets. This advantage position iTSA as a promising technique for target identification in TCM research, especially given the TCM’s intricate nature and multiple mechanisms of action.

By employing the iTSA method, we can identify the molecular targets of TCM. This approach facilitates the establishment of target databases for various TCM herbs, subsequently enabling the construction of disease-target networks. These networks provide a deeper understanding of the mechanisms underlying the therapeutic efficacy of TCM. In our study, we specifically identified the targets of DHI from two TCM herbs Danshen and Honghua, commonly employed in the treatment of cardiovascular and cerebrovascular diseases. This identification provides a theoretical framework for their therapeutic applications, boosting our understanding of how these herbs exert their beneficial effects.

Network pharmacology analysis has become a primary method for studying the effects of TCM on diseases. By constructing networks based on the synergistic interactions between the multi-ingredients and multi-targets of TCM and the disease targets, it can help clarify some potential mechanisms. However, there are limitations to this approach. The main reason is that the targets of the drugs are mostly predicted and thus lack high accuracy. Comparing the targets of DHI identified through our experiments with those predicted by TCMSP (https://tcmsp-e.com/tcmsp.php), we found that only four targets were overlapped, indicating that the other targets may not be direct targets of the drugs (data not shown). Nonetheless, the drug targets predicted by network pharmacology may not be actual binding targets, our PPI network results showed that most of the predicted targets interact with those identified targets via iTSA. It implies that even though the predicted targets may not be direct binding sites, they may still play a role in the overall therapeutic mechanism of the drugs by interacting with the actual targets. Therefore, the results of network pharmacology analysis still possess a certain reference value.

Based on our results, by mixing nine known small molecules and conducting the iTSA experiment, we can obtain targets and rapidly identify the small molecules that bind to these targets. As shown in Fig. 5C, even if we use more small molecules as a mixture, the same results can be achieved. Therefore, for traditional Chinese medicine, by identifying its ingredients, we can quickly pinpoint the targets and match them with their corresponding binding small molecules using our method.

Interestingly, among the 110 targets of DHI, only 35 targets overlap with those known to be related to diseases in the database. This also demonstrates the advantage of TCM in the PDD model. By utilizing our method to obtain target information and corresponding small molecules from TCM herbs that are known to be effective in treating diseases, we may discover novel drug combinations for treating diseases. Another significant application is that once we identify the targets of TCM through this experiment, such as discovering numerous reported targets, we can fish out the corresponding small molecules that have the potential to act as agonists or inhibitors.

The method can also be applied to study TCM in different forms. For standardized samples in which the components are known, our approach enables a relatively rapid determination of global targets, as well as characterization of the active components from TCM through the mixing of known primary components. For decoctions made from natural herbs, we first need to optimize the decoction method and identify their main components using techniques such as mass spectrometry and HPLC. After determining the primary components, we can then apply the method outlined in our article to achieve target identification and active component characterization. In other words, once the compositional information of the traditional Chinese medicine is obtained, subsequent target identification and component fishing can be carried out effectively. Therefore, the method we propose is capable of addressing the challenges posed by such complex decoctions.

## Conclusions

In summary, we successfully identified the targets of DHI, providing valuable information towards understanding the mechanisms underlying DHI. Furthermore, for known major ingredients, utilizing the innovative iTSA approach, we identified the targets and their respective active components. This study not only facilitates the discovery of promising drug candidates and targets, but also plays a pivotal role in unveiling the mechanisms of DHI and advancing the development and modernization of TCM.

## Supplementary Information

Additional file 1.

Additional file 2.

## Data Availability

Data will be made available on request.

## Abbreviations

TCM: Traditional Chinese medicine
iTSA: Isothermal shift assay
DHI: Danhong injection
TDD: Target-based drug discovery
PDD: Phenotypic drug discovery
TPP: Thermal proteome profiling
CETSA: Cellular thermal shift assay
TMT: Tandem mass tag
GO: Gene ontology
KEGG: Kyoto encyclopedia of genes and genomes
PPI: Protein–protein interaction
BP: Biological process
CC: Cellular component
MF: Molecular function
ADK: Adenosine kinase
ALDH1B1: Aldehyde dehydrogenase 1 family member B1
