# Supplementary material for: Development of a new paradigm model for deciphering action mechanism of Danhong injection using a combination of isothermal shift assay and database interrogation
Source: Chin Med. 2024 Oct 5;19:136. doi: 10.1186/s13020-024-01017-6 (PMC11452974; doi:10.1186/s13020-024-01017-6)
Supplement: Supplementary file 1 — Additional file 1. [file 13020_2024_1017_MOESM1_ESM.docx]

| Herb Name | Molecular Formular | Structural Formular |
| --- | --- | --- |
| Rosmarinic acid | C_18_H_16_O_8_ | 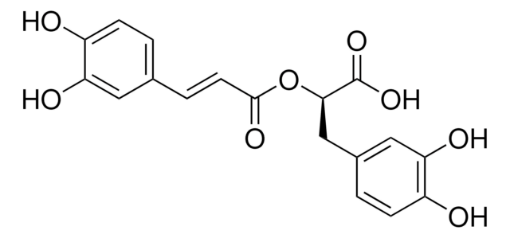 |
| Salvianolic acid A | C_26_H_22_O_10_ | 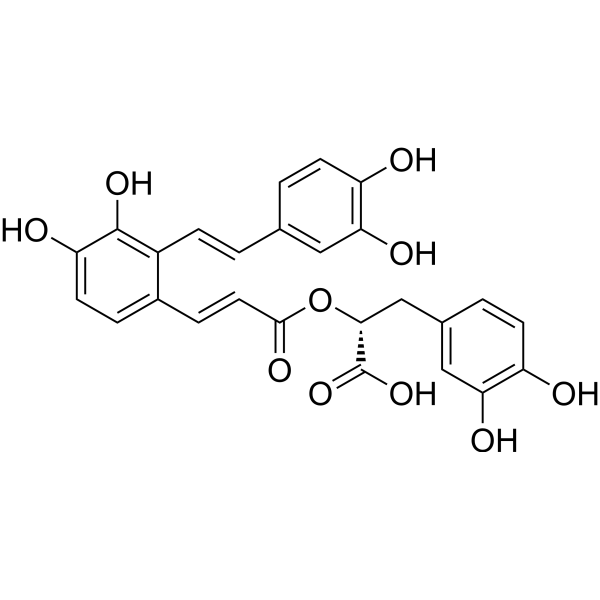 |
| Salvianolic acid B | C_36_H_30_O_16_ | 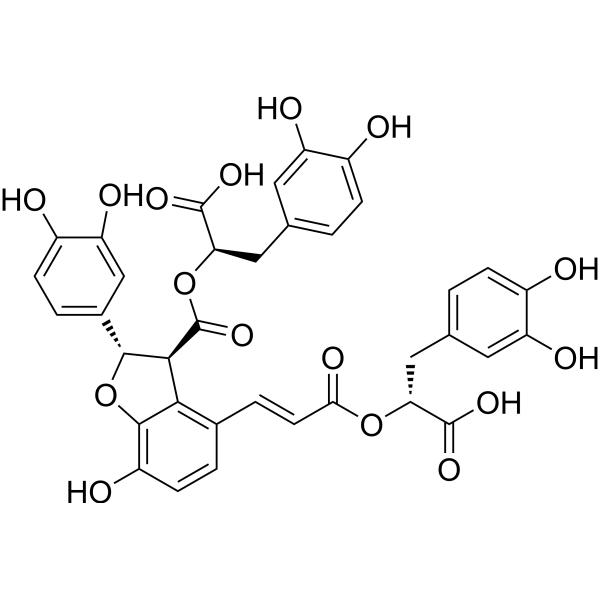 |
| Lithospermic acid | C_27_H_22_O_12_ | 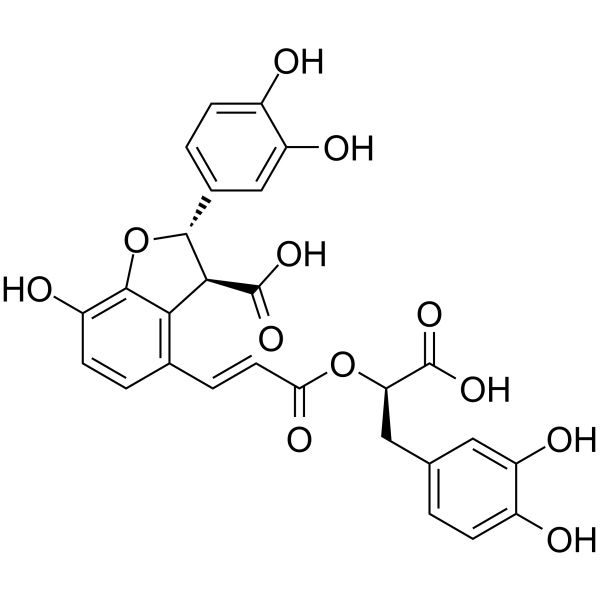 |
| Protocatechu-aldehyde | C_7_H_6_O_3_ | 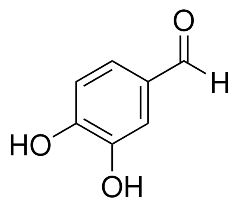 |
| Salvianic acid A | C_9_H_10_O_5_ | 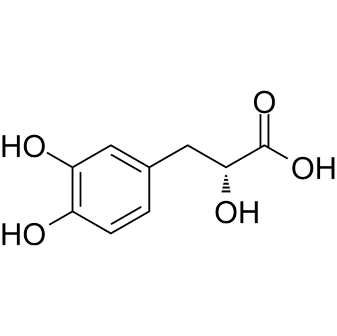 |
| Hydroxysafflor  yellow A | C_27_H_32_O_16_ | 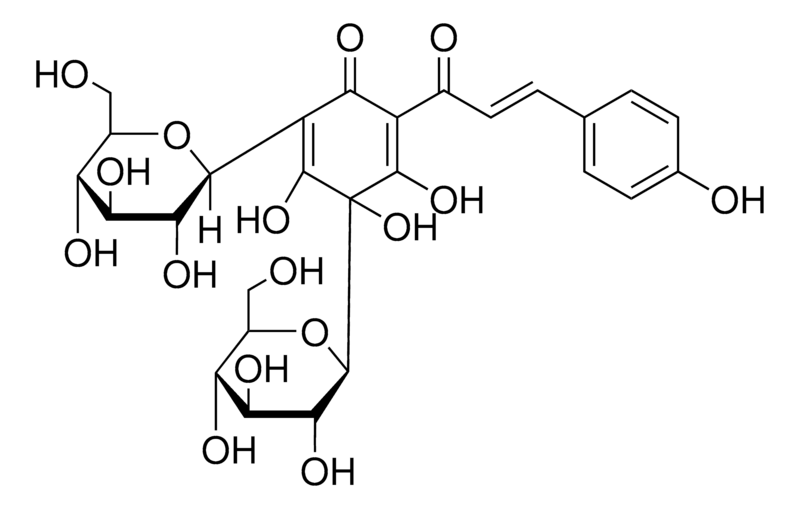 |
| Caffeic acid | C_9_H_8_O_4_ | 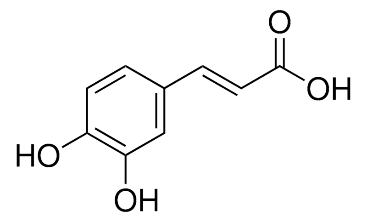 |
| Ferulic Acid | C_10_H_10_O_4_ | 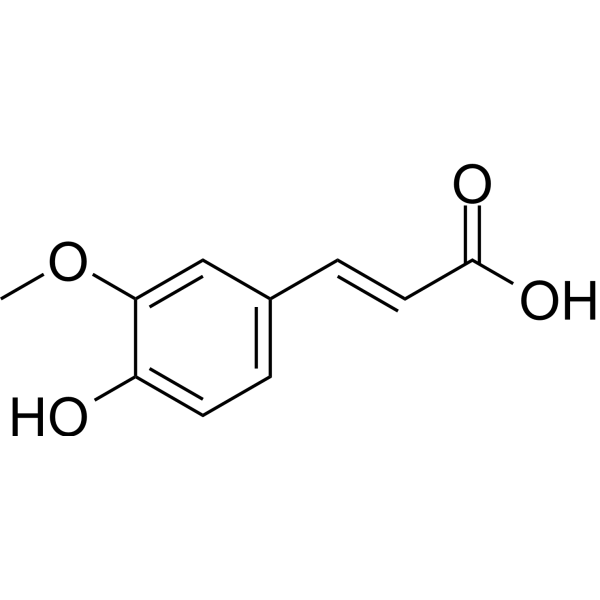 |
